# Application of Physical and Quantum-Chemical Characteristics of Epoxy-Containing Diluents for Wear-Resistant Epoxy Compositions

**DOI:** 10.3390/ma18245643

**Published:** 2025-12-16

**Authors:** Andrii Kulikov, Kostyantyn Sukhyy, Oleksandr Yeromin, Marcel Fedak, Olena Prokopenko, Iryna Sukha, Oleksii Poloz, Oleh Mikats, Tomas Hrebik, Olha Kulikova, Martin Lopusniak

**Affiliations:** 1 Faculty of Manufacturing Technologies with the Seat in Presov, Technical University of Kosice, Bayerova 1, 08001 Presov, Slovakia; marcel.fedak@tuke.sk (M.F.); tomas.hrebik@tuke.sk (T.H.); olha.kulikova@tuke.sk (O.K.); 2 Department of Ecology, Heat Transfer and Labor Protection, Faculty of Mechanical Engineering and Environmental Protection, Ukrainian State University of Science and Technologies, 49000 Dnipro, Ukraine; ksukhyy@gmail.com (K.S.); aoeremin@gmail.com (O.Y.); eprok777@ukr.net (O.P.); irinasuha3@gmail.com (I.S.); ua.apolo@gmail.com (O.P.);; 3 Faculty of Civil Engineering, Institute of Architectural Engineering, Technical University of Kosice, Bayerova 1, 08001 Presov, Slovakia; martin.lopusniak@tuke.sk

**Keywords:** epoxy-containing diluents for epoxy compositions, polyamine hardeners, quantum-chemical calculations, electron gap, surface energy, wetting, spreading

## Abstract

Low-viscosity epoxy-containing diluents are used to reduce the initial viscosity of highly filled, wear-resistant epoxy systems and to improve filler wetting and dispersion. This study determined physical parameters by an atomic-increment approach and electronic descriptors using the Parametric Method 3 (PM3) semi-empirical method. Clear relationships were established between the effective molar cohesion energy and the solubility parameter with van der Waals volume. Linear dependencies were also obtained between the diluent surface tension and spreading coefficients on model high-hardness fillers, including silicon carbide, boron carbide, and normal corundum. The activity of epoxy diluents depends on the lowest unoccupied molecular orbital energy. These diluents influence processing and the final physical and mechanical properties of composites, making their selection critical for strength, hardness, and wear resistance. Computational analysis enables prediction of diluent behavior, reducing experimental time and cost. Integrating physical and quantum-chemical data into epoxy diluent design accelerates the search for optimal components and improves production of durable, high-performance epoxy composites.

## 1. Introduction

To reduce the wear of various equipment operating under the influence of a flow of abrasive materials (hydrocyclones, slurry pumps, slurry pipelines, etc.) in the practice of leading world manufacturers, epoxy wear-resistant composites are used, highly filled with dispersed fillers with high hardness on the Mohs scale, including silicon and boron carbides, normal corundum, etc. [1,2,3]. The introduction of such fillers in high concentrations (300–800 wt. parts per 100 wt. parts of epoxy resin), even in low-viscosity epoxy resins, is associated with technological difficulties in their uniform distribution in the epoxy matrix due to a sharp increase in the viscosity of the compositions. To reduce the initial viscosity of highly filled epoxy compositions, epoxy-containing diluents (EDs) are most often introduced into their composition, such as mono-, di- and polyglycidyl esters of aliphatic alcohols and alkylphenols [4,5], which can interact along with the basis of the compositions—epoxy resin with hardeners—and take part in the modification of polymer chains and the formation of the three-dimensional network and properties of the compositions [5].

Leading world companies have proposed EDs of various chemical structures, the use of which for certain epoxy resins is carried out experimentally and requires significant material costs and time. Therefore, it makes sense to select the necessary EDs with the determination of their predicted parameters—the physical and quantum-chemical characteristics of EDs (van der Waals volume, surface energy, the solubility parameter, dipole moment, and characteristics of the electron structure)—which can be determined by calculation methods using the increments of atoms of these compounds [6] and the energies of molecular orbitals [7], which allows one to reduce experimental costs to achieve the required result. From a quantum-chemical point of view, parameters such as the HOMO (highest occupied molecular orbital) and LUMO (lowest unoccupied molecular orbital) energies, which influence the reactivity of a molecule, are decisive. A smaller difference between the HOMO and LUMO (the so-called “HOMO–LUMO gap”) indicates higher chemical reactivity. Such diluents more easily enter into reactive steps with hardeners or epoxy groups and directly participate in the formation of the polymer network. The electrostatic potentials on the surface of the molecules and the distribution of charge density, which influence the course of nucleophilic and electrophilic reactions during curing, are also important [8,9].

The combination of these properties allows diluents to be classified not only according to their physical effectiveness in reducing viscosity, but also according to their chemical reactivity and influence on the final properties of the cured system. For example, monofunctional diluents (e.g., phenyl glycidyl ether and o-cresyl glycidyl ether) cause chain elongation and a reduction in cross-link density, contributing to increased flexibility, but, at the same time, they can reduce the strength and temperature resistance of the composite. Conversely, difunctional and trifunctional diluents (e.g., diglycidyl ether of 1,4-butanediol and triglycidyl ether of pentaerythritol) create a denser and stronger network, resulting in higher hardness, Tg, and resistance to mechanical wear [10,11].

Modern computational methods, such as Density Functional Theory (DFT) calculations, semi-empirical methods (PM6 and AM1), or fragment-additive approaches (e.g., group contributions to volume or polarity), allow properties to be predicted without the need for synthesis of the substance. These tools allow composite developers to selectively choose suitable EDs even before laboratory verification. For example, Khalina et al. showed that DFT analysis can be used to predict differences in reactivity between different types of diglycidyl ethers and correlate them with cured material yields and mechanical properties [12].

From a practical point of view, the introduction of reactive diluents has a dual significance: on the one hand, they improve the processability of highly filled epoxy compositions, and, on the other hand, they modify the structure and properties of the resulting polymer network. A suitably selected ED can not only reduce the viscosity of the composition to a workable level but also improve the dispersion of fillers and prevent the formation of aggregates or defects in the matrix. This is particularly important in applications with high mechanical or abrasive loads, where microdefects can lead to crack initiation and premature material failure [8]. Although the development of new epoxy systems still relies heavily on experimental methods, the trend is toward hybrid approaches, where computational models are used as a filtering tool to pre-select suitable candidates. In this context, databases of the physical and chemical properties of diluents and resins are being created, which can be used as input for computational simulations and optimization algorithms. Such methodologies significantly accelerate the development process while reducing the costs associated with experimental testing of tens to hundreds of candidate compounds.

Epoxy resin systems can be modified using various additives to tailor their processing and performance characteristics. Low-viscosity epoxy-containing diluents reduce the initial viscosity of epoxy mixtures, significantly improving the wetting and dispersion of fillers, while reactive siloxane diluents can enhance adhesion and workability without compromising mechanical properties at 20 °C [12]. These diluents also influence the cross-linking process through their quantum-chemical parameters, such as the HOMO/LUMO energies and dipole moment, which determine their reactivity with polyamine hardeners. Fillers, including aluminum oxide, quartz, silicon oxide, mica, and graphite, affect shrinkage, thermal conductivity, and long-term stability, with their surface wettability by diluents or the epoxy matrix being a key factor; surface treatment of nano-SiO_2_ fillers further improves matrix compatibility, mechanical strength, and thermal stability, and reduces dielectric losses [13,14,15,16,17]. Resinous modifiers, such as hyperbranched polymers, polysulfides, and phenolic resins, are incorporated to enhance resistance to shock, moisture, and thermal cycling, sometimes participating in cross-linking to modify the polymer structure [18,19,20,21,22,23,24]. Flexibilizers and plasticizers, including polyamides or polyglycol diepoxides, reduce internal brittleness and improve the material’s capacity to absorb mechanical shocks without significantly reducing thermal stability [11]. Finally, reactive elastomers and thermoplastics, such as siloxanes, polysulfones, and polyetherimides, prevent microcrack formation under mechanical stress and contribute to the formation of an internal dispersed phase, which enhances the durability and service life of epoxy composites under cyclic loading [11,14].

In conclusion, the application of the physical and quantum-chemical characteristics of diluents represents a promising direction for the development of epoxy compositions, especially for applications where the requirements for mechanical strength, abrasion resistance, and temperature stability are extremely high. Given the growing demands on the performance and durability of engineering materials, it is expected that the combination of computational chemistry, rheological analysis, and experimental validation will become a standard part of the development of new generations of functional epoxy systems. This approach was used in this study.

## 2. Materials and Methods

Epoxy-containing diluents (mono-, di-, tri-, and tetrafunctional products of world manufacturers (Table 1)) were used individually and also introduced into the industrial epoxy resin KER 828 produced by Kumho P&B Chemicals, Inc., Yeosu-si, Republic of Korea (with Mn = 400 g/mol, an epoxy group content of 21.0 wt.%, and a dynamic viscosity at 25 °C of 14 Pa·s), in an amount of 10 wt. parts per 100 wt. parts of epoxy resin, as recommended by the manufacturers for the manufacture of epoxy compositions. The epoxy compositions were prepared by mechanical mixing of the epoxy resin KER 828 and EDs at a temperature of 60 °C for 5 min. In the filled composites, multidispersed silicon carbide was added to the epoxy matrix after the epoxy diluent, with stirring for 5–7 min. Polyamine hardeners were added to the composites after they had cooled to 20 °C, with stirring for 3–5 min.

The physical–chemical characteristics of the EDs (the van der Waals volume ∑∆Vi, effective molar energy of cohesion Σ∆Ei*, solubility parameter δi, and surface energy σ_d_) were calculated using the atomic increment method using the corresponding formulas given in [6]. The calculations of the ∑∆Vi of the epoxy-containing diluents were carried out by adding increments of the van der Waals volumes ∆Vi of their atoms, Σ∆Ei*, by adding increments ∆Ei*, which characterized the contribution to the cohesion energy of each atom and the type of specific intermolecular interaction. The values of the increments ∆Vi and ∆Ei* were taken from [6]. The calculated values of the surface energy σ_d_ of the epoxy-containing diluents were compared with their experimental values of surface tension σ_s_, determined by the static Wilhelmi method [25] when immersing a platinum plate in an ED.

The quantum-chemical characteristics of the EDs (the energies of the highest occupied molecular orbital (HOMO) and the lowest unoccupied molecular orbital (LUMO), and the dipole moment μ) were calculated with the semi-empirical Parametric Method 3 (PM3) [26,27,28,29,30] using the Chem Office 9.0 Ultra software package, Cambridge Soft 2004. Prediction of the reaction activity of the EDs when interacting with amine hardeners used in curing epoxy compositions using energy-saving technology at “normal” temperature (20 °C) was carried out using the values of the absolute value of the electron gap energy ǀ∆ǀ [31], the absolute difference in the energies of the HOMO of amines and the LUMO of the epoxy-containing diluents. The smaller the absolute value of the electron gap energy was, the more energetically favorable their interaction was [7].

The wetting angle θ of the model surfaces of the fillers used in the wear-resistant epoxy compositions was determined at 20 °C by the “sitting” drop method. The experimental determination of θ was carried out with a special installation [32], which consisted of a digital camera (Canon PC 1562, Tokyo, Japan), a tripod, and a dosing element, which allowed obtaining an image of the profile of an ED drop or an ED composition on the substrate surface. The tripod ensured the coincidence of the optical axis of the camera with the plane of the sample surface. The dosing device provided the same drop parameters and conditions for their application. On the image of the drop, a tangent was drawn at the point of intersection of the drop contour with the sample surface using a graphic editor.

The works of adhesion (W_a_) and cohesion (W_k_) and the spreading coefficient (χ) of the epoxy diluents were calculated using the following formulas [33]:

W_a_ = σ_s_ · (1 + Cos θ),

Wk = 2σ_s_,

χ = W_a_ − W_k_,

where σ_s_ represents the experimental surface tension (or calculated surface energy σ_d_), and θ represents the experimental wetting angle.

Plates made of silicon and boron carbides and normal corundum were used as model wear-resistant surfaces of the fillers.

**Figure 1:**
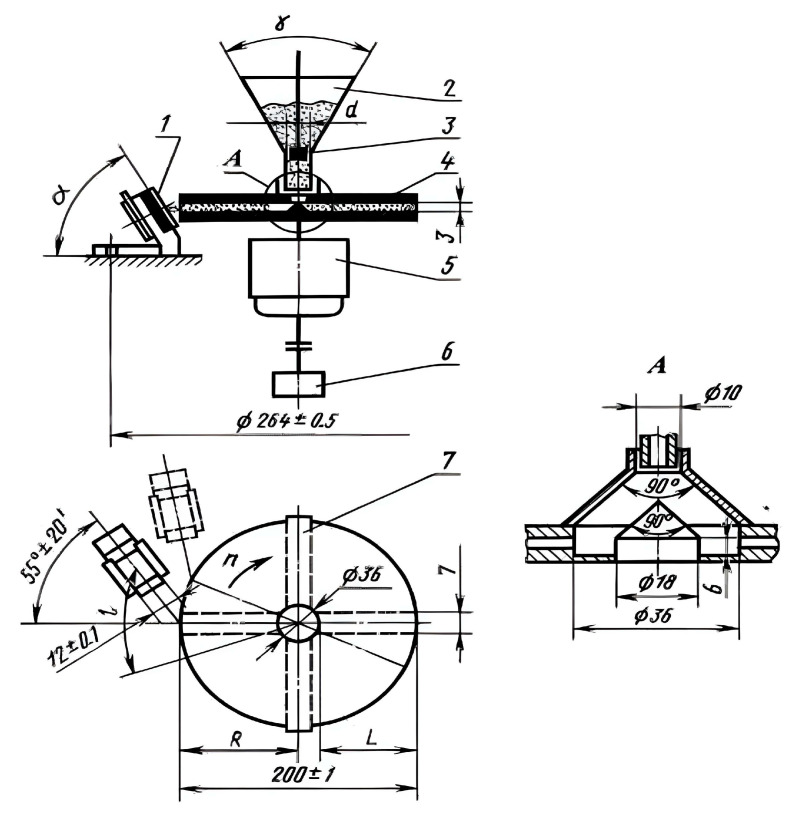
Centrifugal accelerator: 1—sample; 2—hopper; 3—damper; 4—rotor; 5—motor; 6—speed sensor; and 7—rotor radial valve.

The physical–mechanical properties of the epoxy composites were determined in accordance with the current ISO standards, including compressive strength (ISO 604:2003), bending strength (ISO 178:2010), tensile strength (ISO 8256:2017), and Charpy impact strength (ISO 179-1:2022) [34,35,36,37]. Wear tests of the composites were carried out under the action of a gas-abrasive flow (5 kg of river sand with a particle size of 0.5–0.9 mm) under the most severe conditions: a particle flow velocity of 76 m/s and an abrasive attack angle of 15° in a centrifugal accelerator (Figure 1) at a rotor speed of 6000 rpm. The test specimens were manufactured in the form of plates with dimensions of 20 × 15 × 4 mm. The wear of the compositions was characterized by a change in the mass of the samples; the volumetric wear (ΔV) of the samples was calculated by taking into account their density, determined by the hydrostatic method.

## 3. Results and Discussion

**Figure 2:**
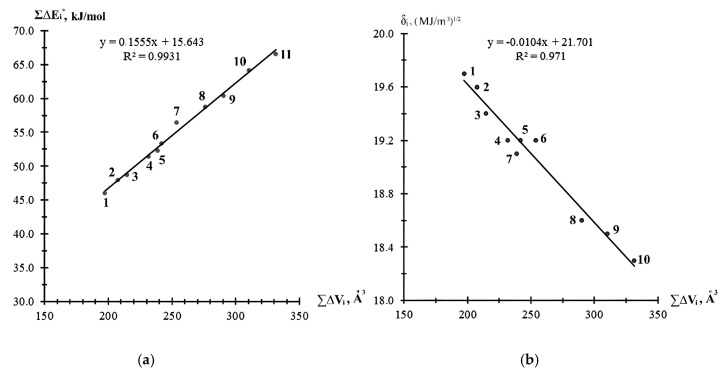
Relationship between effective molar energy of cohesion Σ∆Ei* (**a**) and solubility parameter δi (**b**) with van der Waals volume ∑∆Vi of epoxy-containing diluents: (**a**) 1—RD 3, EPODIL 750, CHS-Epoxy RR 800, 2—RD 17, 3—RD 14, 4—RD 18, 5—CL12, 6—CHS-Epoxy RR430, 7—RD 11, 8—CHS-Epoxy RR 330, TCM AGE, RD 24 (*n* = 12), 9—CHS-Epoxy RR 690, RD 20, EPOSIR 8103, 10—CHS-Epoxy RR 330, TCM AGE, RD 24 (*n* = 14), and 11—CL16; (**b**) 1—RD 3, EPODIL 750, CHS-Epoxy RR 800, 2—RD 17, 3—RD 14, 4—RD 18, 5—CHS-Epoxy RR430, 6—RD 11, 7—CL12, 8—CHS-Epoxy RR 690, RD 20, EPOSIR 8103, 9—CHS-Epoxy RR 330, TCM AGE, RD 24 (*n* = 14), and 10—CL16.

**Figure 3:**
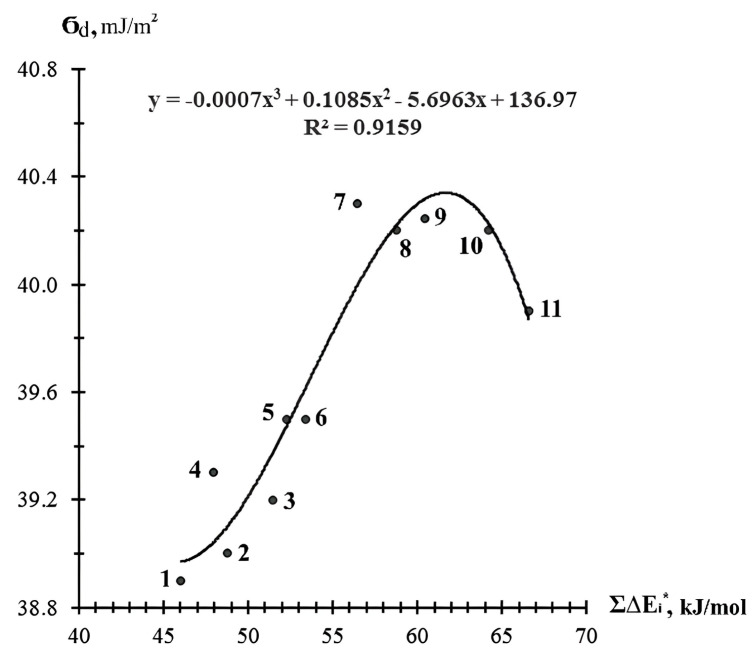
Dependence of the surface energy σ_d_ of epoxy-containing diluents on their effective molar energy of cohesion Σ∆Ei*: 1—RD 3, EPODIL 750, CHS-Epoxy RR 800; 2—RD 14; 3—RD 18; 4—RD 17; 5—CL12; 6—CHS-Epoxy RR430; 7—RD 11; 8—CHS-Epoxy RR 330, TCM AGE, and RD 24 (*n* = 12); 9—CHS-Epoxy RR 690, RD 20, and EPOSIR 8103; 10—CHS-Epoxy RR 330, TCM AGE, and RD 24 (*n* = 14); and 11—CL16.

Figure 2 and Figure 3 present the dependencies of the physical–chemical characteristics calculated by the atomic increment method, including the effective molar energy of cohesion Σ∆Ei*, the solubility parameter δi, and surface energy σ_d_ from the chemical structure of the EDs of world manufacturers. The data presented show that the δi and Σ∆Ei* of the epoxy-containing diluents were related to their van der Waals volume by clear linear dependencies (Figure 2a,b), with a high value for the pair correlation coefficient (>0.97), which allowed the specified values to be calculated directly from the value of the van der Waals volume ∑∆Vi using the defined equations, without performing the necessary, more complex calculations. The surface energy σ_d_ of the epoxy-containing diluents was most correctly related to the values of their effective molar cohesion energy Σ∆Ei* (Figure 3).

The effective molar energy of cohesion and surface energy are related as they reflect molecular interactions in epoxy diluents. For diluents with a more branched structure (CHS-Epoxy RR 690; RD 20; EPOSIR 8103), the surface energy should be higher. The higher the surface energy, the higher the cohesion energy (Figure 3), and the higher the physical and mechanical performance of the cured epoxy compositions should be [38].

It should be noted that the calculated values of the surface energy σd of the epoxy diluents (Table 2) differed slightly from their experimental values of surface tension σ_s_ (deviations Δ did not exceed ±5%) and were sufficiently reliable for use in subsequent calculations.

**Figure 4:**
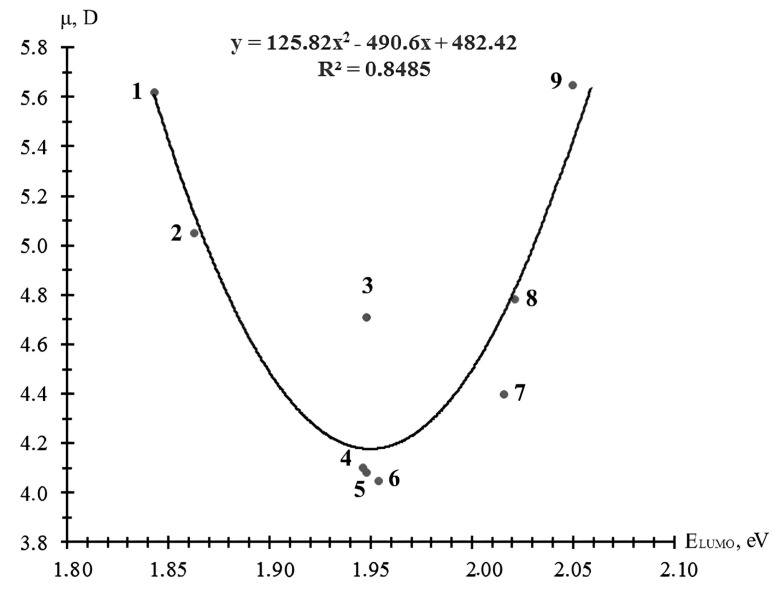
Dependence of the dipole moment μ of epoxy-containing diluents on their LUMO energy: 1—CL12; 2—CL16; 3—RD 3, EPODIL 750, CHS-Epoxy RR 800; 4—CHS-Epoxy RR430; 5—CHS-Epoxy RR 330, TCM AGE, and RD 24; 6—RD 17; 7—RD 11; 8—CHS-Epoxy RR 690, RD 20, and EPOSIR 8103; and 9—RD 14.

The different numbers of low-polar hydrocarbon and polar glycidyl groups in the EDs had a more significant effect on the values of their dipole moments μ (Figure 4), which was due to the spatial arrangement, number, and order of polar groups, and the distribution of electron density in the molecules. The smallest dipole moments were characteristic of the monoglycidyl esters containing increased amounts of hydrocarbon groups, and the largest ones were characteristic of the di- and polyglycidyl esters. The most correct dependence of the dipole moment on the energy of the LUMO of the epoxy-containing diluents was parabolic (Figure 4).

The relationships presented in Figure 2 and Figure 3, which are described by the proposed equations, allowed for preliminary calculations of the complex parameters of epoxy diluents (surface energy and dipole moment) and their use in predicting the wetting and spreading processes of diluents on the filler surface in filled composites, the formation of composite structures, and the molecular design of diluents.

According to the data in Table 3, the wetting angle of the epoxy-containing diluents of model high-hardness on the Mohs scale of the filler surfaces depended on their surface energy and the energy of the interaction with the substrate. The best wetting (by the minimum values of the wetting angle θ) of these surfaces was observed for the triglycidyl ether of trimethylolpropane CHS-Epoxy RR690 and the monoglycidyl ethers CHS-Epoxy RR430, TCM AGE, and CHS-Epoxy RR330. It should be noted that the triglycidyl ether of trimethylolpropane from different manufacturers (CHS-Epoxy RR690, Czech Republic, and EPOSIR 8103, Italy) was characterized by slightly different values of surface tension (38.0 mN/m and 40.6 mN/m, respectively) and different abilities to wet the specified substrates, which was probably due to the different amounts of impurities in them. All considered, the EDs better wetted the model high-hard surfaces than the epoxy matrices of the compositions, epoxy resin KER 828 (the wetting angles of the fillers with epoxy resin were as follows, in degrees: boron carbide—67, silicon carbide—65, and normal corundum—75), and their wetting of the substrates improved with a decrease in their surface tension. When 10 wt.% ED was added to epoxy resin KER 828, the wetting angle of the model surfaces with this epoxy composition also changed linearly.

**Figure 5:**
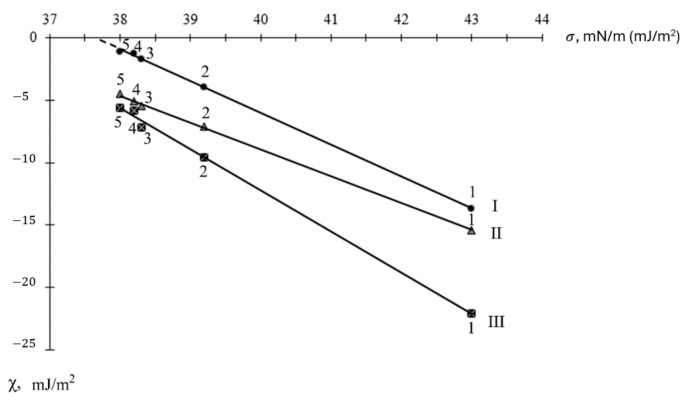
Dependence of the spreading coefficients χ of epoxy-containing diluents on the surfaces of model high-hardness fillers on their surface tension σ: 1—DEG-1, 2—CHS-Epoxy RR330, 3—TSM AGE, 4—CHS-Epoxy RR430, and 5—CHS-Epoxy RR690; model surfaces of fillers: I—silicon carbide, II—boron carbide, and III—normal corundum.

The improvement of the EDs spreading on the high-hardness surfaces of the fillers (Table 3) occurred according to the classical scheme, due to an increase in the adhesion energy and a decrease in the cohesion energy (mainly due to a more significant decrease in the cohesion energy of these compounds). The ED spreading coefficients χ on these surfaces were related to their experimentally determined surface tension σ by clear linear dependencies (with pair correlation coefficients >0.99 (Figure 5)), which were characterized by the following equations for the fillers:

Boron carbide: y = −2.149x + 77.023 (R2 = 0.9990);(1)

Silicon carbide: y = −2.550x + 95.953 (R2 = 0.9996);(2)

Normal corundum: y = −3.264x + 118.24 (R2 = 0.9930),(3)

where x is the surface tension of the ED, and y is the spreading coefficient χ.

The obtained dependencies allowed predicting the surface tension values for the complete spreading of these compounds (χ > 0) on the surfaces of the substrates. Thus, for complete spreading on the surface of silicon carbide, the ED must have a surface tension σ < 37.8 mN/m (mJ/m^2^).

The considered fillers (Figure 5) had different surface energies and, accordingly, different interactions with the epoxy diluents, which was reflected in the work of adhesion and, accordingly, in the spreading coefficients of the epoxy diluents on their surfaces.

For the rational selection of existing and new EDs during the curing of epoxy compositions at “normal” temperature, their activity in reactions with aliphatic polyamines and the alicyclic polyamine N-aminoethylpiperazine (EAP), the chemical structure and characteristics of which are given in [31], was established by the values of the absolute value of the electron gap energy ǀ∆ǀ (Table 4). Analysis of the data in Table 4 showed that in reactions with the presented polyamines, the most active was the triglyceride ether of glycerol (with a LUMO energy of 1.843 eV), and the least active was the diglycidyl ether of neopentyl glycol (with a LUMO energy of 2.042 eV). In order of increasing activity in reactions with the EDs, the polyamine hardeners presented in Table 4 are arranged in the following sequence: hexamethylenediamine (HMDA) < tetraethylenepentamine (TEPA) < triethylenetetramine (TETA) < diethylenetriamine (DETA) < N-aminoethylpiperazine (AER).

The same sequence of activity of these polyamines according to quantum-chemical calculations was confirmed experimentally during the curing of ED-20 epoxy resin, based on the characteristic temperature of the exothermic reaction interaction of the resin epoxy groups with the amine groups of the hardeners, where the more active the polyamine, the higher the temperature of the exothermic reaction [31]. A relationship with a sufficiently high pair correlation coefficient (0.977) was established [31] between the maximum temperature of the exothermic curing reaction and the absolute values of the electron gap energy ǀ∆ǀ between the LUMO of ED-20 epoxy resin and the HOMO of the polyamines, which made it possible to predict the activity of epoxy diluents in reactions with polyamine hardeners based on the calculated values of ǀ∆ǀ or on the experimentally determined maximum temperature of the exothermic curing reaction.

Thus, the results of the calculations of the physical and quantum-chemical characteristics of the epoxy-containing diluents and the dependencies obtained on their basis were the basis for molecular design to determine their optimal structures when introduced into epoxy compositions.

The epoxy groups of the diluents were reaction centers that, together with the epoxy groups of the main matrix of the compositions—epoxy resin KER 828—reacted with the amine groups of the hardener polyethylenepolyamine—Polyamine B (AkzoNobel, Sweden)—at 10 wt. parts per 100 wt. parts of the resin with the diluent. In this case, monoglycidyl esters (CHS-Epoxy RR330 and CHS-Epoxy RR430) could only connect the epoxy chain, modifying it. The diglycidyl ether ERODIL 750 and the triglycidyl ether CHS-Epoxy RR690 entered into a reaction of elongation of the growing chain and cross-linking. The triglycidyl ether, due to its three epoxy groups, was able to be incorporated into the growing chain and simultaneously ensure its cross-linking with the formation of a denser three-dimensional network.

Indeed, as evidenced by the sol–gel analysis data (Table 5), the lowest content of the sol fraction and, accordingly, the highest cross-linking coefficient was provided to the epoxy composites by the triglycidyl ether CHS-Epoxy RR690, and the lowest by the monoglycidyl ether CHS-Epoxy RR 330, which contains a long hydrocarbon chain. The long (C12-C14) hydrocarbon chain of this diluent provided improved wetting and spreading of the compositions on the high-hardness surfaces, performing the function of a surfactant, and did not participate in the reactions of the three-dimensional network formation.

**Figure 6:**
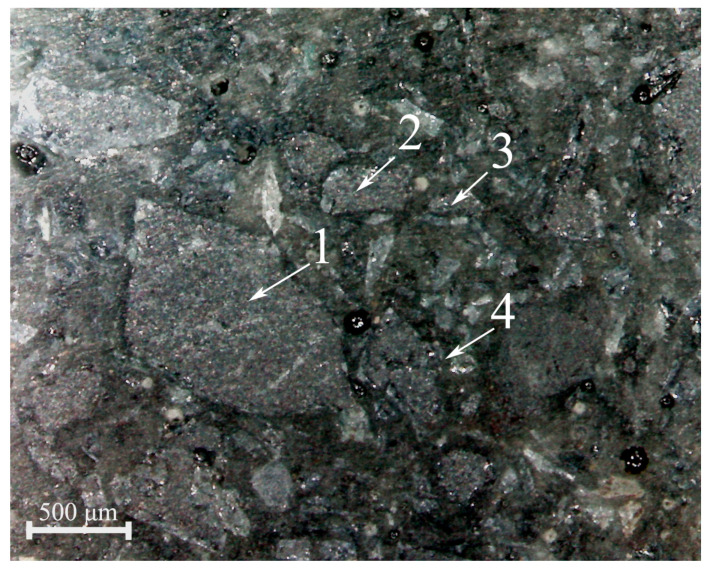
Optical image of the dispersed structure (×100) of polydisperse silicon carbide in a wear-resistant epoxy composite of optimal composition: 1—1600–2000 μm; 2—400–500 μm; 3—125–200 μm; and 4—5–7 μm.

Depending on the cross-linked network formed in the unfilled epoxy composites with the epoxy diluents, their physical and mechanical properties and wear resistance varied (Table 5). For the filled composites, the elastic strength properties and wear resistance were determined by both the formed cross-linked network and the structure of the interfacial layer, the dispersed phase of the filler [39]. In this case, an important role was played by the processes of wetting and spreading the epoxy binder (epoxy resin + epoxy diluent) over the filler surface, and its adhesive interaction with the filler surface [40], forming a strong interfacial layer and a dispersed structure with a high packing density of filler particles (Figure 6). A comparison of the effects of two epoxy diluents (CHS-Epoxy RR 690 and DEG-1), which differed in their parameters of wetting and spreading over the surface of silicon carbide, with close values of the cross-linking coefficients of the composites, showed the advantage of using CHS-Epoxy RR 690 (especially for the most sensitive characteristics of composites, such as tensile strength, impact strength, and gas-abrasive wear, to the adhesive interaction with the surface of the fillers, which largely depend on the course of the processes of wetting and spreading of binders over the surfaces of the fillers).

## 4. Conclusions

Using atomic increments and the semi-empirical Parametric Method 3 (PM3), calculations of the main physical, quantum, and chemical characteristics of epoxy-containing diluents of leading world manufacturers for epoxy compositions were performed depending on their functionality (mono-, di-, tri-, and tetrafunctional), the branching of their molecular structures, and the number of low-polar hydrocarbon groups. The calculations were used for the targeted use of the epoxy-containing diluents in epoxy compositions, taking into account their reactivity when interacting with polyamine hardeners, and their surface properties during wetting and spreading on the model surfaces of high-hardness fillers (silicon and boron carbides and normal corundum) of wear-resistant epoxy compositions.

A relationship was established (with pairwise correlation coefficients greater than 0.97) between the effective molar energy of cohesion and the solubility parameter with the van der Waals volume of epoxy-containing diluents. Dependencies were obtained that allow for preliminary calculations of the complex parameters of epoxy diluents (surface energy and dipole moment) and their use in predicting the wetting and spreading processes of diluents on the filler surfaces of filled composites, the formation of composite structures, and the implementation of the molecular design of diluents.

The linear dependencies of the spreading coefficients of epoxy-containing diluents on the studied model surfaces of high-hardness fillers and their surface tension were established with high reliability (the pair correlation coefficient was more than 0.99), which allows predicting the surface tension values of epoxy-containing diluents for optimizing the wetting and spreading processes.

It was found that in the reactions of the interaction with the studied polyamines, the activity of the epoxy-containing diluents was determined by the values of their LUMO energy. The most active was the triglyceride ether of glycerin with the minimum LUMO energy value (1.843 eV), and the least active was the diglycidyl ether of neopentyl glycol with the maximum LUMO energy value (2.042 eV). The polyamine hardeners were arranged in the following sequence, depending on their HOMO energy values, in terms of increasing their activity: hexamethylenediamine (HMDA) < tetraethylenepentamine (TEPA) < triethylenetetramine (TETA) < diethylenetriamine (DETA) < N-aminoethylpiperazine (AEP).

The given calculation dependencies were applied when selecting an effective epoxy diluent (CHS-Epoxy RR690) for unfilled and multidispersed silicon carbide-filled wear-resistant epoxy compositions.

## Figures and Tables

**Table 1:** Characteristics of epoxy diluents (EDs).

Chemical Formula (Brand) of ED	Manufacturer (Country)	Epoxy Equivalent, g	Dynamic Viscosity at 25 °C, mPa·s
Glycidyl ether 2-ethylhexane (RD 17) 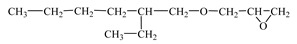	IPOX CHEMICALS GmbH (Laupheim, Germany)	210–230	2–4
Alkyl glycidyl ether C8–C10 (CHS-Epoxy RR 430) 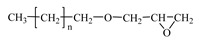 n = 8–10	SPOLCHEMIE (Ústí nad Labem, Czech Republic)	270–313	1–6
Alkyl glycidyl ether C12–C14 (CHS-Epoxy RR 330; TCM AGE; RD 24) 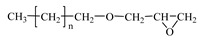 n = 12–14	SPOLCHEMIE (Ústí nad Labem, Czech Republic); Triune Chemicals and Materials (Shoushansi Township, China); IPOX CHEMICALS GmbH (Laupheim, Germany)	270–330	5–10
Diglycidyl ether 1,4-butanediol (RD 3; EPODIL 750; CHS-Epoxy RR 800) 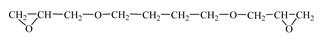	IPOX CHEMICALS GmbH (Laupheim, Germany); Air Products (Allentown, PA, USA); SPOLCHEMIE (Ústí nad Labem, Czech Republic)	130–145	12–22
Diglycidyl ether dimethanolcyclohexane (RD 11) 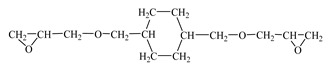	IPOX CHEMICALS GmbH (Laupheim, Germany)	165–185	60–90
Diglycidyl ether neopentyl glycol (RD 14) 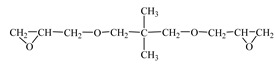	IPOX CHEMICALS GmbH (Laupheim, Germany)	150–160	15–25
Diglycidyl ether 1.6-hexanediol (RD 18; CHS-Epoxy RR 700) 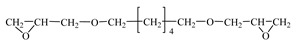	IPOX CHEMICALS GmbH (Laupheim, Germany); SPOLCHEMIE (Ústí nad Labem, Czech Republic)	147–161	15–25
Diglycidyl ether diethylene glycol (DEG-1) 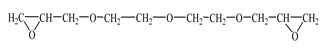	JSC “NIIKHIMPOLIMER” (Perm, Russia)	140–150	65–75
Triglycidyl ether trimethylolpropane (CHS-Epoxy RR 690; RD 20; EPOSIR 8103) 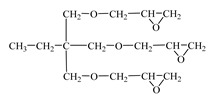	SPOLCHEMIE (Ústí nad Labem, Czech Republic); IPOX CHEMICALS GmbH (Laupheim, Germany); SIR industriale (Italy)	140–150	120–180
Triglycidyl ether glycerin (CL 12) 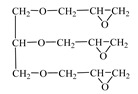	IPOX CHEMICALS GmbH (Laupheim, Germany)	140–150	160–200
Tetraglycidyl ether pentaerythritol (CL 16) 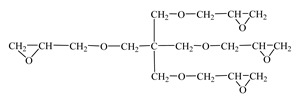	IPOX CHEMICALS GmbH (Laupheim, Germany)	156–170	900–1200

**Table 2:** Comparative assessment of calculated (σ_d_) and experimental (σ_s_) values of surface energy (surface tension) of epoxy diluents.

ED	σ_s_, mN/m	σ_d_, mJ/m^2^	Δ, %
CHS-Epoxy RR 690	38.0	39.6	+4.2
CHS-Epoxy RR 430	38.2	39.3	+2.9
TCM AGE	38.3	40.2	+4.9
CHS-Epoxy RR 330	39.2	40.2	+2.5
DEG-1	43.0	41.3	−4.0

**Table 3:** Wetting angle (Ѳ), work of cohesion (W_k_) and adhesion (W_a_), and spreading coefficient (χ) values of EDs on the surfaces of model high-hardness fillers.

Brand of ED	σ_s_, mN/m	W_k_, mJ/m^2^	Boron Carbide			Silicon Carbide			Normal Corundum		
			Ѳ, degree	W_a_, mJ/m^2^	χ, mJ/m^2^	Ѳ, degree.	Wa, mJ/m^2^	χ, mJ/m^2^	Ѳ, degrees	Wa, mJ/m^2^	χ, mJ/m^2^
CHS–Epoxy RR 690	38.0	76.0	28	71.5	−4.5	14	74.9	−1.1	28	70.4	−5.6
CHS–Epoxy RR 430	38.2	76.4	30	71.3	−5.1	15	75.1	−1.3	32	70.6	−5.8
TCM AGE	38.3	76.6	31	71.1	−5.5	17	74.9	−1.7	37	68.9	−7.7
CHS–Epoxy RR 330	39.2	78.4	35	71.3	−7.1	26	74.4	−4.0	41	68.8	−9.6
DEG-1	43.0	86.0	50	70.6	−15.4	47	72.3	−13.7	61	63.9	−22.1

**Table 4:** Energies of molecular orbitals of EDs and absolute values of the energy of the electron gap ǀ∆ǀ in their interaction with industrial polyamine hardeners.

Brand of ED	Energies of Molecular Orbitals ED, eV		E_HOMO_ of Polyamines, eV				
			HMDA (−9.402)	DETA (−9.289)	TETA (−9.305)	TEPA (−9.316)	AER (−9.083)
	HOMO	LUMO	ǀ∆ǀ, eV				
CL 12	−10.857	1.843	11.245	11.132	11.148	11.159	10.926
CL 16	−10.722	1.863	11.265	11.152	11.168	11.179	10.946
CHS-Epoxy RR 430	−10.986	1.946	11.348	11.235	11.251	11.262	11.029
RD 3, EPODIL 750, and CHS-Epoxy RR 800	−10.839	1.948	11.35	11.237	11.253	11.264	11.031
CHS-Epoxy RR 330, TCM AGE, and RD 24	−10.967	1.948	11.35	11.237	11.253	11.264	11.031
RD 17	−10.831	1.954	11.356	11.243	11.259	11.270	11.037
RD 18 and CHS-Epoxy RR 700	−10.782	1.954	11.356	11.243	11.259	11.270	11.037
RD 11	−10.918	2.016	11.418	11.305	11.321	11.332	11.099
CHS-Epoxy RR 690, RD 20, and EPOSIR 8103	−10.645	2.021	11.423	11.310	11.326	11.337	11.104
RD 14	−10.680	2.042	11.444	11.331	11.347	11.358	11.125

**Table 5:** Parameters of the cross-linked structure, physical and mechanical properties, and wear resistance during gas-abrasive wear with sand of 0.5–0.9 mm (medium velocity of 76 m/s) of cured 10 wt. parts of Polyamine B (20 °C × 24 h + 100 °C × 3 h) and epoxy compositions (10 wt. parts of diluent per 100 wt. parts of epoxy resin KER828).

Diluent	Content of Sol Fraction, %	Cross-Linking Coefficient	Impact Toughness, kJ/m^2^	Bending Strength, MPa	Tensile Strength, MPa	Compressive Strength, MPa	Gas-Abrasive Wear (∆V·103, cm^3^) During Abrasive Attack at Angle of 15°
Unfilled compositions							
CHS-Epoxy RR 330	1.35	7.59	7.1	95	15.3	140	12.8
CHS-Epoxy RR 430	0.50	13.14	8.9	93	17.9	145	8.6
ERODIL 750	0.28	17.90	14.0	91	21.8	155	7.2
CHS-Epoxy RR 690	0.19	21.94	16.8	87	23.1	163	6.4
Filled composites (600 wt. parts of multidispersed silicon carbide of composition with a particle size (μm) of 5–7 (25%) + 125–200 (28%) + 400–500 (15%) + 1600–2000 (32%) per 100 wt. parts of epoxy resin KER828 with diluent)							
CHS-Epoxy RR 690	0.21	20.87	3.3	61	15.6	127	6.9
DEG-1	0.22	20.37	3.0	58	14.2	121	8.2

## Data Availability

The original contributions presented in this study are included in this article. Further inquiries can be directed to the corresponding author.
